# Real-Time Wireless Tumor Tracking in Navigated Liver Resections: An Ex Vivo Feasibility Study

**DOI:** 10.1245/s10434-022-11364-z

**Published:** 2022-02-23

**Authors:** Roeland Eppenga, Wout Heerink, Jasper Smit, Koert Kuhlmann, Theo Ruers, Jasper Nijkamp

**Affiliations:** 1 Department of Surgical Oncology, The Netherlands Cancer Institute, Amsterdam, The Netherlands; 2 Nanobiophysics Group, Faculty TNW, University of Twente, Enschede, The Netherlands

**Keywords:** Liver resection, Surgical navigation, Wireless navigation, Electromagnetic tracking, Tumor motion tracking, Nonpalpable lesion

## Abstract

**Background:**

Surgical navigation systems generally require intraoperative steps, such as intraoperative imaging and registration, to link the system to the patient anatomy. Because this hampers surgical workflow, we developed a plug-and-play wireless navigation system that does not require any intraoperative steps. In this ex vivo study on human hepatectomy specimens, the feasibility was assessed of using this navigation system to accurately resect a planned volume with small margins to the lesion.

**Methods:**

For ten hepatectomy specimens, a planning CT was acquired in which a virtual spherical lesion with 5 mm margin was delineated, inside the healthy parenchyma. Using two implanted trackers, the real-time position of this planned resection volume was visualized on a screen, relative to the used tracked pointer. Experienced liver surgeons were asked to accurately resect the nonpalpable planned volume, fully relying on the navigation screen. Resected and planned volumes were compared using CT.

**Results:**

The surgeons resected the planned volume while cutting along its border with a mean accuracy of − 0.1 ± 2.4 mm and resected 98 ± 12% of the planned volume. Nine out of ten resections were radical and one case showed a cut of 0.8 mm into the lesion. The sessions took approximately 10 min each, and no considerable technical issues were encountered.

**Conclusions:**

This ex vivo liver study showed that it is feasible to accurately resect virtual hepatic lesions with small planned margins using our novel navigation system, which is promising for clinical applications where nonpalpable hepatic metastases have to be resected with small resection margins.

The preferred treatment of colorectal liver metastases (CRLM) is surgical resection, resulting in highest overall survival rates.^1,2^ In the past, resections were performed with a minimal resection margin of 10 mm, but over the years it has been indicated that smaller margins of  ≥ 1  mm do not result in lower survival rates or higher local recurrence rates.^3–5^ The same applies to margins of less than 1 mm when detaching CRLM from major intrahepatic vessels.^6^ This expands surgical options and more cases can be considered for surgery.

Smaller margins demand for better awareness of lesion borders during surgery. This can be challenging, for example, during laparoscopic procedures but also when operating on nonpalpable lesions, lesions with complex shapes, or large deformable lesions.^7,8^ In these cases, intraoperative ultrasound (IOUS) often is used for assessing lesion borders.^9^ However, two-dimensional (2D) IOUS images can be difficult to relate to the 3D lesion information from preoperative imaging and lack usability when operating on vanishing or isoechoic lesions.^10–12^

As an alternative or addition to using IOUS, navigation techniques can be used.^13^ In a common navigation setting, the surgeon has a tracked object (e.g., pointer or forceps) of which the location is visualized “real-time” on a screen, relative to the patient’s anatomy in preoperative imaging. Anticipating on what is visualized while moving the tracked object, the surgeon can locate the lesion in the actual anatomy and assess its borders.

The preoperative images presented on the navigation screen are snapshots of the anatomy at the time that they were acquired. If navigation techniques fully rely on these images, lesion border assessment will be inaccurate due to peroperative anatomical motion and deformation. Especially during liver surgery, a lesion can shift up to several centimeters due to breathing, tissue deformation, and surgical manipulation.^14^ Most techniques compensating for this effect try to estimate the new lesion location by detecting and modeling the anatomical changes.^13,15,16^ However, a more straight-forward approach is to track the lesion motion directly, using trackers implanted near or in the lesion. Studies on lesion tracking using electromagnetic (EM) trackers have shown promising results.^17–20^ Most of these EM trackers are wired, which requires intraoperative implantation and subsequent intraoperative imaging to locate the tracker relative to the lesion after which the system can be calibrated. These intraoperative steps consume intraoperative time and hamper surgical workflow.

This can be avoided by using wireless EM trackers. Until now, the Calypso^®^ System (Varian Medical Systems Inc., Palo Alto, CA), designed for radiotherapy, is the only clinically cleared EM tracking system using wireless trackers (transponders).^21^ In previous work, we showed the technical possibilities for using this system in a surgical setting and obtained promising results for navigated lumpectomies during a study on breast phantoms.^22,23^ By design, the Calypso system is limited to tracking three transponders simultaneously, all used for real-time lesion tracking in radiotherapy. This limitation also was clear in the breast phantom study, where other tools had to be tracked with an additional tracking system. However, recent work showed that accurate lesion position and orientation tracking also is possible using only two transponders, leaving the third transponder for tracking a surgical tool.^24^ This allows for a simple straightforward workflow with minimal added time and no need for intraoperative imaging.

In this ex vivo study, we assessed whether this simpler workflow can work in clinical practice and whether it is feasible to resect a nonpalpable volume, consisting of a virtual lesion plus 5 mm margin, using only the wireless EM technology of the Calypso system for tracking tool and lesion. Feasibility will indicate nonpalpable hepatic lesions with small planned surgical margins can be accurately resected using this system. Navigated ex vivo virtual lesion resections were performed on large human hepatectomy specimens. Surgical accuracy was assessed by measuring the 3D surgical margins on computed tomography (CT) imaging.

## Materials and Methods

### Study Design

Ten large hepatectomy specimens were selected for this study. All contained enough healthy tissue for resecting a volume of about 40 mm in diameter, without interfering with pathological analysis. On each specimen, an ex vivo resection of a virtual lesion with nonpalpable borders was conducted by an experienced liver surgeon, using our novel wireless navigation setup. This study was performed in accordance with the Declaration of Helsinki and received approval from our Institutional Review Board. According to Dutch law, written, informed consent from patients was not required.

### Navigation System

**Fig. 1:**
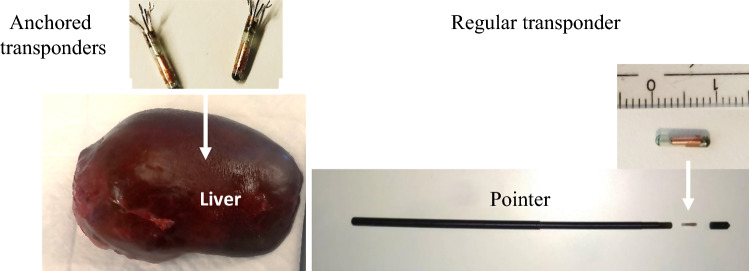
Wireless transponders used in this study. One regular transponder of 8 × 1.85 mm in size is mounted inside the cylindrical pointer (right). Two anchored transponders, of similar dimensions, are inserted into the liver specimen (left)

The navigation system used in this study comprised the Varian Calypso tracking system and in-house developed navigation software with a navigation interface. The Varian Calypso system is an electromagnetic (EM) tracking system, able to track three, 8 × 1.85 mm, wireless transponders (Fig. 1).

Its Tracking Array (TA) generates EM fields that excite the transponders upon which they emit signals back to the TA. Using these signals, the Calypso system tracks the transponders with < 0.5 mm accuracy when they are within the tracking area of 140 × 140 × 190 mm, starting at 80 mm above the TA.^22,25^ In our setup, two transponders were used for lesion tracking. The third transponder was used for tracking the in-house developed cylindrical pointer of 5 × 153 mm, mounted inside and close to the pointer tip and in-line with the pointer axis (Fig. 1).

After the transponders for lesion tracking were preoperatively implanted, a planning CT was acquired to locate the transponders relative to the anatomy. Once these locations were indicated by the user, the navigation software automatically registered these locations to the corresponding locations measured by the Calypso system. This linked the transponder tracking to the planning CT and therefore also the lesion, planned surgical margin and liver contour, after which the navigation system was ready to use. Navigation was simply done by moving the pointer and anticipating on what was shown in the navigation interface.

**Fig. 2:**
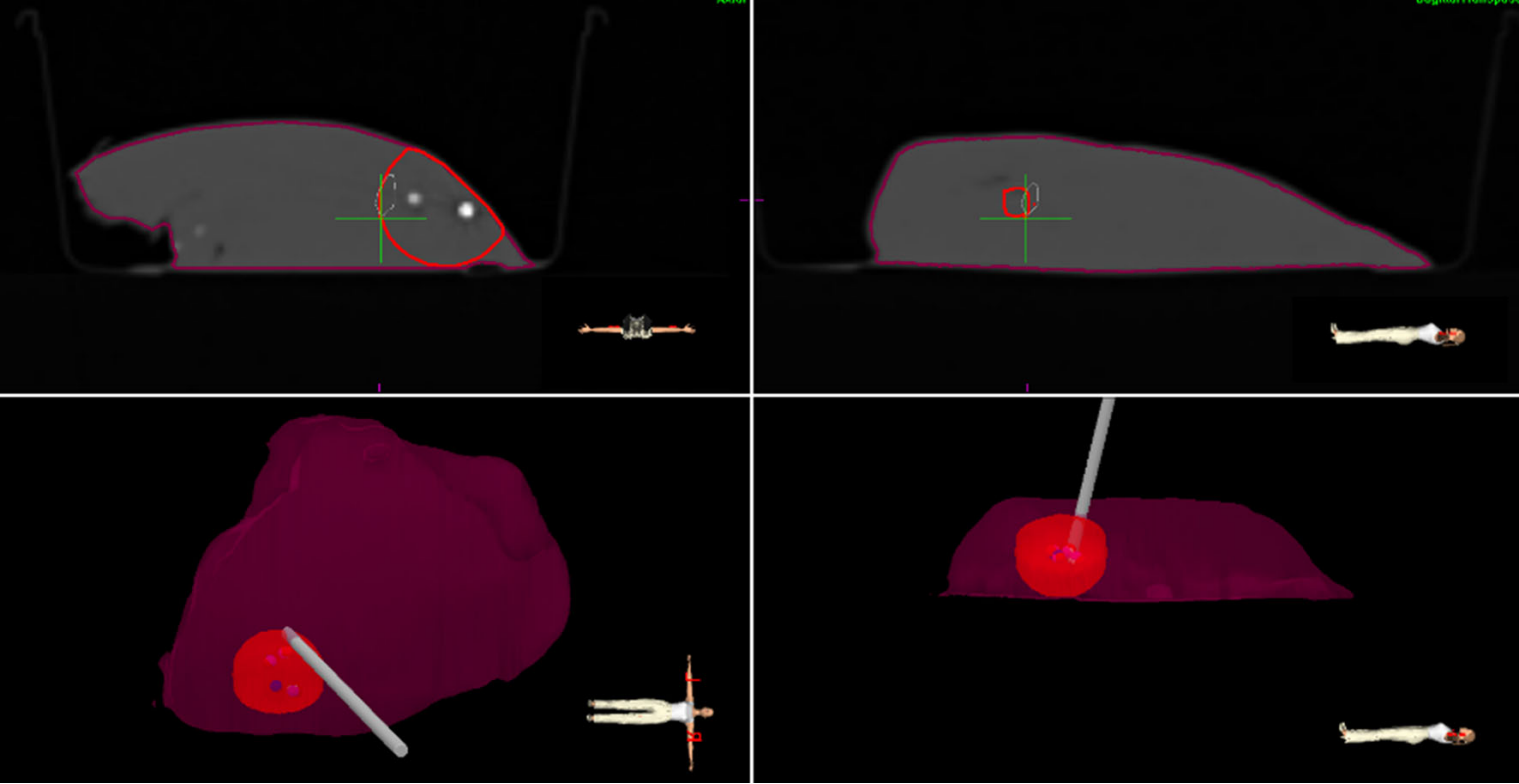
Navigation interface. Bottom views show the real-time position of the pointer (grey rod) relative to the 3D model of the hepatectomy specimen, from coronal (left) and sagittal (right) perspective. Top views are the axial and sagittal CT slices corresponding to the real-time pointertip location, indicated with a green crosshair. In all four views, maroon represents the specimen contour and red the planned resection volume

The navigation interface had four views (Fig. 2). Two views showed the pointer relative to the hepatectomy specimen in 3D. The other two views showed the axial and sagittal CT slices of the specimen, corresponding to the real-time pointer tip location. All views were updated 8 times per second. Only the planned resection volume (PRV) was shown in these views, but the surgeons knew the lesion was 5 mm underneath the PRV surface.

### Preparation Workflow

**Fig. 3:**
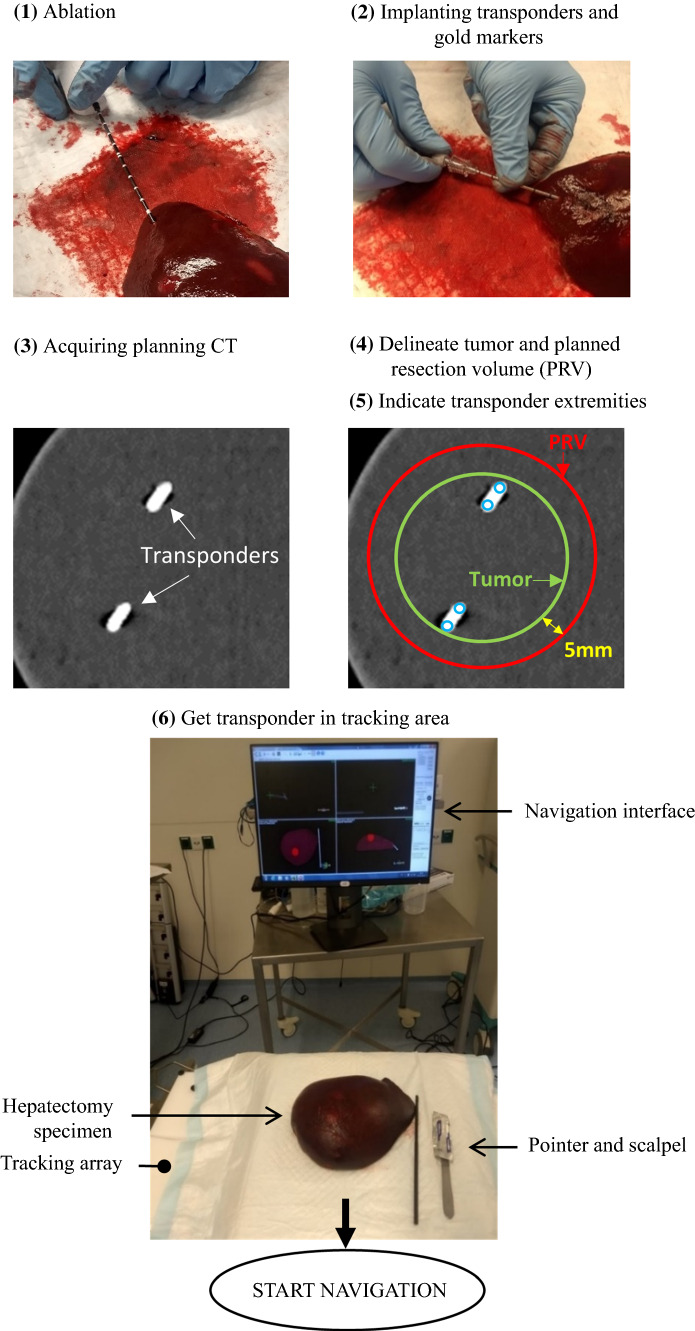
The 6-step workflow to prepare the navigation. Each step number is indicated between round brackets. The blue open circles in step 5 indicate the transponder extremities

The 6-step workflow to prepare each navigated resection is illustrated in Fig. 3. In step 1, one of the researchers performed a microwave ablation on the area selected for the navigated resection. The purpose of this ablation step was to stiffen the area and thereby minimize tissue deformation that would strongly reduce the accuracy of surgical margin assessment in the postprocessing. Due to the stiffness of the ablated tissue, the surgeon could roughly locate the PRV through palpation. However, even though the ablated area enclosed the PRV, the ablated area had an undefined shape and size. This means that the borders of the ablated area had an unknown relationship with the borders of the PRV or lesion. Therefore, to assess the nonpalpable PRV and lesion borders within the ablated area, the surgeon had to fully rely on the navigation interface.

Directly after step 1, in step 2, two anchored transponders were implanted inside the hepatectomy specimen, using a 13G coaxial biopsy needle.^26^ The transponders were implanted with approximately 25 mm distance and a considerable angle between them.^24^ To assess migration of the transponders, three VISICOIL gold markers of 0.35 mm by 3 ± 1 mm (RadioMed Corporation, Bartlett, TN) were implanted in the same area as the two transponders.^27^

Steps 3 to 5 were required to prepare the navigation interface (Fig. 2). First, a CT image of the liver specimen was acquired with 1mm slice thickness (step 3). Then, within this planning CT, a virtual lesion was delineated as a sphere encompassing the transponders and gold markers, and the PRV was delineated isotropically around the lesion with a 5 mm margin (step 4). Finally, step 5 was to manually indicate the transponders extremities within the planning CT, after which this CT was automatically registered with the transponder tracking.

After completing these 5 steps, the hepatectomy specimen was ready for the navigated resection and positioned with the transponders inside the TA tracking area (step 6).

#### Navigated Resection

The surgeon primarily used a surgical scalpel for cutting the tissue and a tracked pointer for navigation (Fig. 3). Assistance was provided when requested, e.g., for handling the tissue with retractors or spreaders, for zooming in and out the navigation views and for rotating the 3D model or adjusting its transparencies. Each surgeon approached the navigated resection in two steps. First, the pointer was used to localize the PRV in the specimen and to determine the first cuts on the liver surface. Then, during the actual resection, the surgeon constantly alternated the pointer with the scalpel to ensure the resection was along the PRV border.

### Postprocessing and evaluation

**Fig. 4:**
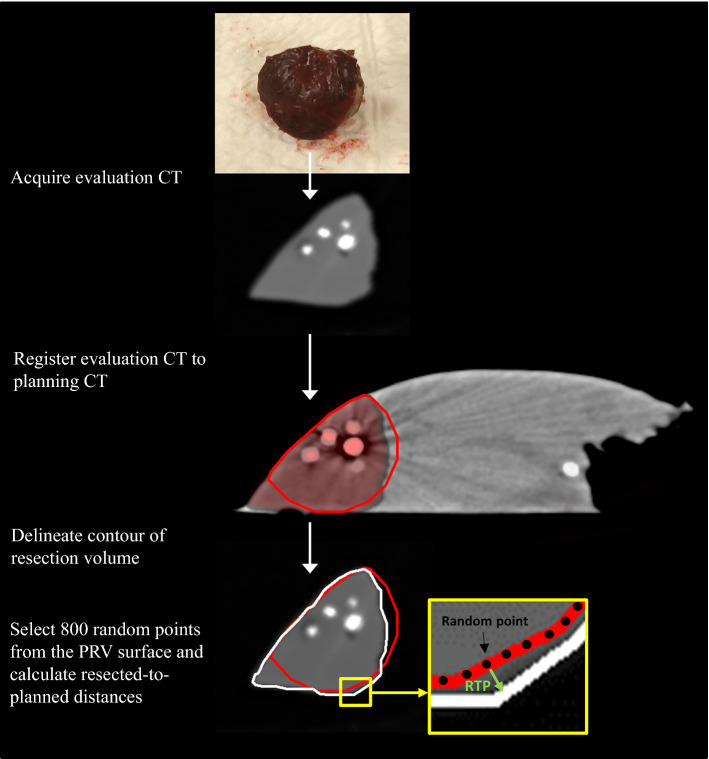
Postprocessing workflow. The red line is the PRV delineation. The red shade is the overlay of the evaluation CT registered on the planning CT. The white line is the auto-segmented contour of the resected volume. In the bottom right illustration the green arrow indicates the resected-to-planned border distance (RTP) for one of the 800 random points. In this example, and all other hepatectomy specimens, the PRV was generally a dented sphere, because the specimen contour was used as a border for this delineation

After the ex vivo resection, a CT image was acquired of the resected volume (Fig. 4). This evaluation CT was registered to the planning CT, based on transponder and gold marker locations visible in both CT images. If the transponders visually appeared to have migrated, registration was based on only the gold markers. The actual resected volume was automatically segmented and compared with the PRV. Eight hundred random points were automatically selected from the PRV surface inside the liver. For each point, the shortest distance to the resected volume contour was calculated, where a negative distance indicated a cut through the surface of the PRV toward the lesion, and a positive distance a too wide excision. These resected to planned border distances (resected-to-planned distances, illustrated with the green “RTP” arrow in Fig. 4) were the primary outcome measure of this study. The secondary outcome measure was the relative resection volume, i.e., resected volume divided by planned volume.

## Results

All ten sessions were completed without any considerable issues regarding the entire workflow. The average registration accuracy of the planning CT with the transponder tracking was 0.4 mm root-mean-square-error. Transponder migration, relative to the gold markers, was minimal, 0.2 ± 0.2 mm (±SD).

Four liver surgeons participated in this study. Two of them each treated more than 20 patients using a previous version of this navigation system and already practiced with this new wireless version on a phantom and two ex vivo human hepatectomy specimens.^28,29^ The other two surgeons were new to this type of navigation system and only observed one of the sessions performed by a “trained” surgeon. The trained surgeons performed eight of ten sessions and the untrained surgeons each only one.

**Fig. 5:**
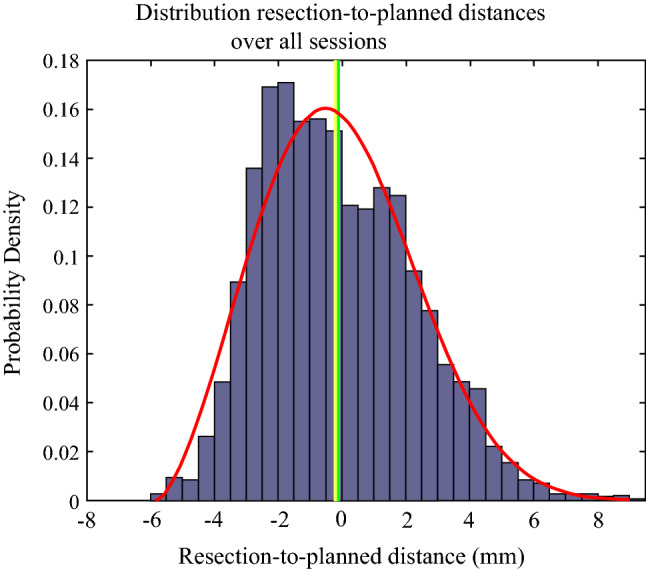
Probability density distribution of all resected-to-planned distances over all sessions, where negative resected-to-planned distances indicate cuts below the planned volume border and positive distances indicate cuts outside this border (i.e., a too wide excision). Each histogram bar represents the percentage of resected-to-planned distances falling within that particular range. The red line represents the corresponding Gaussian distribution (shifted Weibull). The green and yellow vertical lines represent the mean and median resected-to-planned distance over all sessions, respectively

**Fig. 6:**
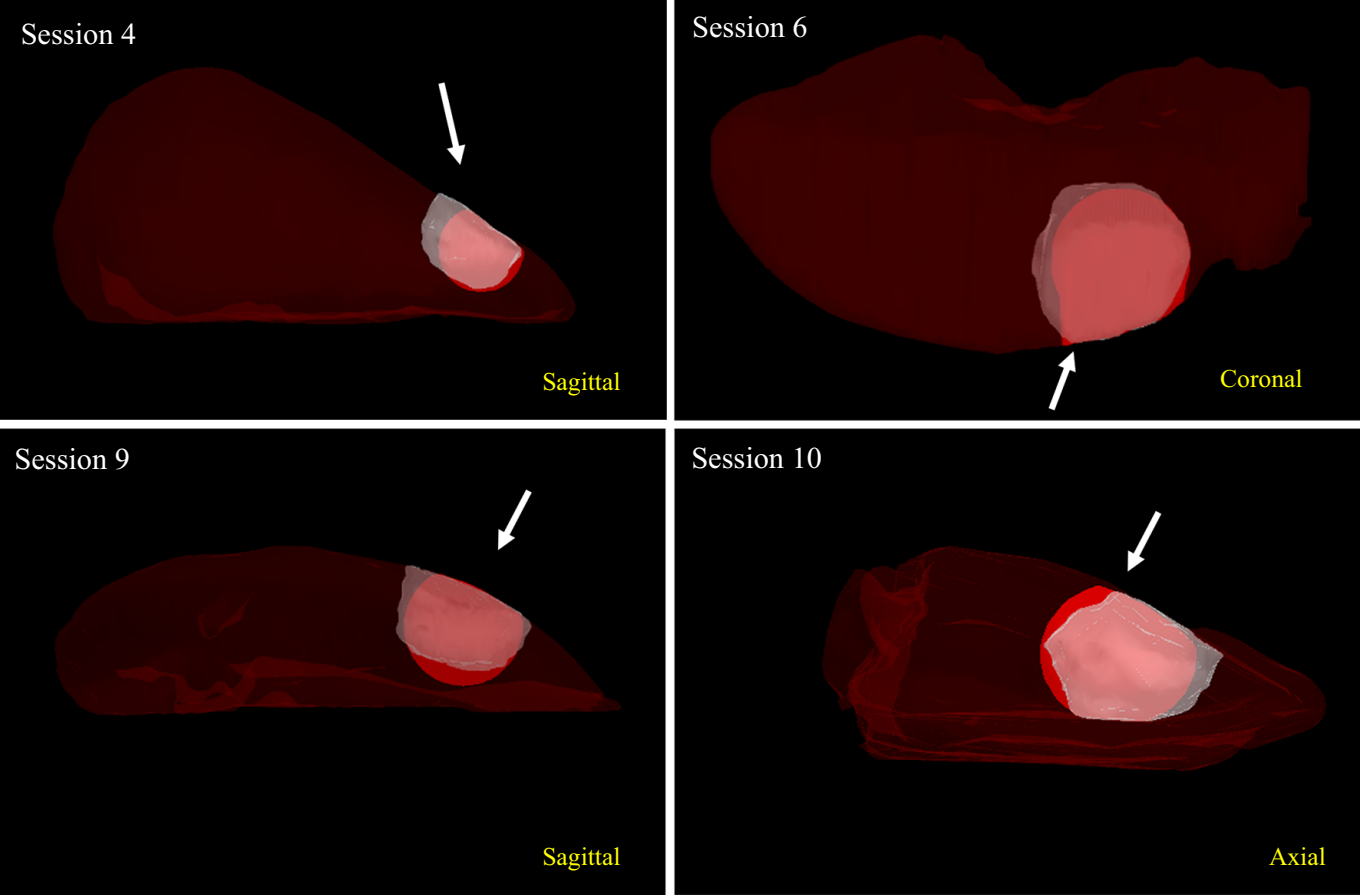
3D illustrations of four typical resections, where session 9 resulted in an irradical resection. Maroon represents the intact hepatectomy specimen contour, red the planned resection volume and white the resected volume contour. In yellow text, the viewing perspective on the 3D model, where the sagittal perspective represents how the specimen was oriented in front of the surgeon. The white arrows indicate the default viewing direction of the surgeon in that specific session. The session numbers are in correspondence with Table 1

All surgeons needed approximately 10 min to complete the navigated resection. The main results are summarized in Fig. 5 and Table 1. The mean PRV diameter was 33 ± 3.0 mm. Surgeons cut accurately along the PRV border with a mean resected-to-planned distance of − 0.1 ± 2.4 mm, where 95% of the distances were between − 4.0 and 4.8 mm. The resected-to-planned distances followed a slightly right-skewed distribution with an overall median of  − 0.4 mm, close to the overall mean of − 0.1 mm (Fig. 5). Median was slightly below the mean in individual cases as well, expect for session 4 where the distribution was strongly right-skewed. In session 9, there was an irradical resection because of a 0.8 mm cut through the lesion border at the deepest point within the specimen. The resection result of session 4 and 9 are illustrated in Fig. 6, alongside two other resection results typical for this study.

**Table 1:** Results for resected-to-planned distances and relative volume per session, with the corresponding planned resection volume (PRV) diameter

Session	Surgeon	Resected-to-planned distances (mm)					Relative volume (%)	PRV diameter (mm)
		Min	Max	Median	Mean	SD		
1	T1	− 3.7	4.9	0.0	0.0	2.0	100	36
2	T1	− 3.0	6.5	− 0.2	0.0	1.8	101	40
3	T1	− 3.8	5.1	− 0.2	− 0.1	2.1	99	30
4	T1	− 3.4	9.1	− 1.4	− 0.3	3.0	94	30
5	T1	− 3.4	5.5	− 0.3	0.0	2.1	100	30
6	T2	− 2.3	6.3	1.3	1.5	1.9	129	35
7	T2	− 3.5	5.9	− 0.9	− 0.3	2.2	94	30
8	T2	− 4.5	6.0	− 1.0	− 0.5	2.6	92	32
9	U1	− 5.8	7.6	− 0.8	− 0.6	2.8	89	35
10	U2	− 4.6	4.9	− 1.2	− 0.9	2.4	83	32
	Overall	− 5.8	9.1	− 0.4	− 0.1	2.4	98 ± 12	33 ± 3.0

Surgeons are divided in two trained (T1 and T2) and two untrained (U1 and U2) surgeons

The average relative resection volume was 98 ± 12% over all sessions, with one outlier of 129% by a trained surgeon (session 6, Fig. 6) and somewhat smaller resections by the untrained surgeons.

## Discussion

In this prospective ex vivo liver study, we presented a novel navigation system that uses electromagnetic (EM) wireless transponders. Surgeons were asked to use this system for navigated resection of a virtual hepatic lesion with a planned resection volume (PRV). The entire navigation workflow was straight-forward as expected and showed no considerable technical issues. This study indicates that this simpler workflow, with no intraoperative actions, such as imaging or registration, can work in clinical practice. The results also indicated that, using this navigation setup, it is feasible to resect CLRMs with nonpalpable borders with a small 5 mm planned margin. The participating liver surgeons resected the PRV while staying within a mean distance of − 0.1 ± 2.4 mm from the PRV border. This is a remarkable result, given that the exact borders of the PRV and lesion were not palpable (or otherwise distinguishable from surrounding tissue) and the surgeons thus solely relied on the navigation.

This navigation setup incorporates lesion motion without intraoperative registration or imaging interpretation. To our knowledge, this combination of features is unique in literature. To compare our results with studies using other navigation techniques, these studies also should report on pathological margins versus planned margins. Only two of these studies were found. The first conducted five in vivo navigated open liver resections using IOUS with EM lesion tracking.^30^ A wired EM tracker was intraoperatively implanted for tracking an area where the PRV (lesion plus 10 mm margin) was close to a “no-touch” area of major vascular structures. Their navigation interface visualized the tracked PRV and sent acoustic signals when the tip of the dissection instrument entered the no-touch area. Their resected-to-planned distances were − 2 mm on average, ranging from − 6 to 4 mm, comparable to our findings. However, their approach required line-of-sight for tracking the dissection instrument and added an average of 20 min to the intraoperative time. The other in vivo study registered preoperative imaging with IOUS by swabbing important salient features with a pointer and then match the pointer tip data with the surface of a preoperative model.^31^ This was repeated after considerable liver motion or deformation. In seven nonanatomical liver resections, the average resected-to-planned distance was 4.2 ± 2.8 mm. These numbers imply less accurate resections than in our study, which could be due to the re-registrations after considerable deformations that could have been avoided with intraoperative lesion tracking. Both studies indicated navigated liver lesion resections to be feasible, but their intraoperative workflow consumed intraoperative time and was complex compared to our proposed workflow. Using our system, even surgeons with limited navigation experience were able to complete the task with an accuracy comparable to experienced surgeons, indicating our system also is intuitive.

The results of the current study are in line with our previous study on breast phantoms.^23^ There, surgical navigation with wireless lesion tracking was shown to be beneficial over radioactive seed localization. Especially for more complex lesion shapes, constant feedback about lesion position and orientation seemed to be key. In this study, an optical tracking system was used for pointer tracking, requiring line-of-sight and thereby strongly reducing flexibility and efficiency. The update on that navigation setup, presented in this manuscript, does not require an additional tracking system and thereby simplifies intraoperative workflow. Comparing the results of the breast phantom study with this ex vivo study confirms recent findings that the updated navigation system provides similar accuracy.^24^

Even though the overall median and mean resection-to-planned distance were almost 0 mm, the distribution ranged from approximately − 6 to + 9 mm (Fig. 5). This could be due to tissue deformation while performing the resection. The PRV deforms when force is applied to it, while the PRV visualized on the screen keeps its same rigid shape. Then, the actual distance of the PRV border to the transponders is smaller than depicted on the navigation screen. The opposite also applies when force is applied on the healthy tissue side. The examples in Fig. 6 indicate that this could indeed have caused some of the inaccurate resection parts. Practicing PRV border assessment while minimizing surgeon-induced tissue deformation by his hands, surgical tools, or pointer may improve results. In addition, the relative movement between the transponders can be used as a measure for deformation. In the navigation interface, this can then either be visualized or generate a warning sign can when the intertransponder movement crosses a certain threshold.

The PRV in this study was defined as the lesion volume with 5 mm margin. This was smaller than the 10 mm margin in our breast phantom study, because we expected more accurate resections and with a smaller margin less hepatectomy specimens had to be rejected due to insufficient amount of healthy tissue. The planned 5 mm was insufficient during one irradical resection (0.8 mm below the lesion border) of an untrained surgeon. Even though practice and aforementioned technical updates may improve results, we do not claim a 5 mm planned margin to be sufficient for safe resection. The nature of a controlled ex vivo study makes it not fully representative for clinical practice. Prospective clinical studies are needed to assess the actual required margin. This study had a couple of limitations. Transponders were inside the lesion, which not always resembles clinical practice. Simulating situations with different transponder configurations relative to the lesion is part of future research. The ex vivo setting allowed for maximal accessibility of the liver, whereas this is limited in an in vivo or clinical setting, especially during laparoscopic procedures. Less accessibility may affect the accuracy of navigated resections. Because of this ex vivo setting, it also was not possible to perform resections with a more commonly used cautery tool. The ablation step, in the preparation workflow, changed the natural tissue elasticity. Nonablated liver tissue has a higher elasticity, increasing the deformation during PRV resection. Rigid anatomical information displayed on the navigation screen may then be less reliable, affecting the navigation accuracy. Simultaneously, the ablation step added great value to this study, because it allowed for resecting a virtual tumor with nonpalpable borders and thus create a task where the surgeon solely relied on the navigation When alternating between using the pointer and scalpel, the feedback provided by the navigation interface cannot be directly applied which may introduce inaccuracies. Tracking the scalpel or a cautery tool, instead of the pointer, may be a solution.^23^ However, the surgeon must be aware that the navigation interface is not reliable while cutting, because then the tissue deforms. No learning curve could be assessed, because the untrained surgeons only performed one session. Even though the trained surgeons performed slightly better on average, it was not clear if this was due to their (limited) training with this particular system or their experience with similar systems. More training is thought to improve results for all participating surgeons, but further research is required.

The workflow illustrated in Fig. 3 is similar to how it can be implemented in clinical practice, excluding the ablation. In the days before surgery, the transponders can be implanted transcutaneously under ultrasound guidance at the radiology department. They can be implanted in or near the lesion, depending on the risk for tumor seeding. After implantation, a planning CT is acquired, on which the lesion is delineated with the help of a radiologist. In this step, diagnostic MR imaging also can be registered to improve the lesion delineation. Subsequently, a PRV can be defined as indicated by the surgeon with the possibility to choose variable margins relative to the lesion. The only required action in the OR is to make sure the transponders are within the tracking area of the TA, and then the navigation can start. This entire workflow can be translated to other areas with moving lesions, such as rectum and breast.^20,23^

## Conclusion

This study shows the potential of accurately resecting hepatic lesions with nonpalpable borders and small planned surgical margins, using a novel navigation system that tracks lesion and tool with wireless electromagnetic trackers. Preparation for these navigations is straight-forward and can entirely be done preoperatively, facilitating plug-and-play navigation in the operating room. The presented findings are promising for the in vivo research that will follow.
